# Clinical impact of Prostate-Specific Membrane Antigen Positron Emission Tomography (PET) on intensification or deintensification of advanced renal cell carcinoma management

**DOI:** 10.1007/s00259-023-06380-4

**Published:** 2023-08-18

**Authors:** Shivanshan Pathmanathan, Arsalan Tariq, Adam Pearce, Handoo Rhee, Samuel Kyle, Sheliyan Raveenthiran, David Wong, Rhiannon McBean, Phillip Marsh, Steven Goodman, Nattakorn Dhiantravan, Rachel Esler, Nigel Dunglison, Anojan Navaratnam, John Yaxley, Paul Thomas, David A. Pattison, Jeffrey C. Goh, Chun Loo Gan, Matthew J. Roberts

**Affiliations:** 1 Department of Medical Oncology, Royal Brisbane and Women’s Hospital, Brisbane, Queensland Australia; 2 Department of Urology, Royal Brisbane and Women’s Hospital, Brisbane, Queensland 4029 Australia; 3 Faculty of Medicine, The University of Queensland, Brisbane, Queensland Australia; 4 Wesley Urology Clinic, The Wesley Hospital, Brisbane, Queensland Australia; 5 Department of Urology, Princess Alexandra Hospital, Brisbane, Queensland Australia; 6 Department of, Medical Imaging, Royal Brisbane and Women’s Hospital, Brisbane, Queensland Australia; 7 Department of Nuclear Medicine, Royal Brisbane and Women’s Hospital, Brisbane, Queensland Australia; 8 Department of Urology, Redcliffe Hospital, Brisbane, Queensland Australia; 9 I-MED Radiology, The Wesley Hospital, Brisbane, Queensland Australia; 10 Department of Diagnostic Radiology, Princess Alexandra Hospital, Brisbane, Queensland Australia; 11 Centre for Clinical Research, The University of Queensland, Brisbane, QLD Australia

**Keywords:** Prostate specific membrane antigen, Positron emission tomography, Renal cell carcinoma, Staging, Management

## Abstract

**Purpose:**

There is an emerging role of the use of Prostate-Specific Membrane Antigen (PSMA) Positron Emission Tomography (PET) in renal cell carcinoma. Herein, we report our experience in use of PSMA PET in recurrent or metastatic renal cell carcinoma (RCC).

**Methods:**

A retrospective analysis of all patients who underwent PSMA PET for suspected recurrent or de-novo metastatic RCC between 2015 and 2020 at three institutions was performed. The primary outcome was change in management (intensification or de-intensification) following PSMA PET scan. Secondary outcomes included histopathological correlation of PSMA avid sites, comparison of sites of disease on PSMA PET to diagnostic CT and time to systemic treatment.

**Results:**

**Supplementary Information:**

The online version contains supplementary material available at 10.1007/s00259-023-06380-4.

51 PSMA PET scans were performed in 48 patients for assessment for recurrence on conventional imaging (*n*=35, 73%) or de-novo metastatic disease (*n*=13, 27%). PSMA PET changed management in 11 patients (11/35, 31%) with recurrent disease, and 3 patients (3/13, 23%) with de-novo metastatic disease. For recurrent disease, better delineation of suspected recurrence led to treatment intensification in 23% (8/35; additional/altered resection, 17%; systemic therapy rather than resection, 6%), and treatment de-intensification in 9% (3/35; surveillance rather than systemic therapy, 3%; resection, 3%; and biopsy, 3%). For de-novo metastatic disease, PSMA PET lead to treatment intensification in 23% (3/13) of patients (systemic from local therapy, 15%; different systemic therapy, 8%). 30 patients underwent biopsy of PSMA avid sites, with clear cell RCC histology in 87%, as well as de-differentiated sarcomatoid RCC (7%), carcinoid (3%) and urothelial cancer (3%).

## Conclusions

PSMA PET provided clinically useful information for patients with diagnostic CT staging, leading to a change in management in 29% of patients with advanced RCC. Prospective research is warranted to further investigate the oncological outcome of PSMA PET determined management in advanced RCC.

## Introduction

Kidney cancer is the 7^th^ most diagnosed malignancy in the developed world, with renal cell carcinoma (RCC) encompassing approximately 75% [1]. Unfortunately, 20-30% of patients are diagnosed with metastatic disease at diagnosis, and up to 40% develop recurrence despite adequate surgery for local disease [2, 3]. The management of advanced RCC is dictated by radiological imaging for staging. Oligo-metastatic RCC for instance, can be managed with local therapies such as metastasectomy or radiation, which can delay the requirement for systemic therapy and improve survival outcomes [4, 5]. More extensive recurrence, or de-novo metastatic disease is more typically managed with systemic therapy such as tyrosine kinase inhibitors, immunotherapy or their combination [6]. Computerised tomography (CT) scan is routinely used for delineation of extent of disease, or staging, and assessing response to treatment. However, CT is limited by standard criteria for accurate assessment of nodal disease, and in the detection of small volume local recurrence and metastasis [7].

Prostate-specific membrane antigen (PSMA) positron emission tomography (PET) uses a radioactive tracer, that binds to PSMA protein, which has better diagnostic accuracy than CT and bone scan for prostate cancer due to PSMA protein over-expression [8]. Interestingly, PSMA protein is also expressed in the neo-vasculature of other solid tumours including RCC, with tracers binding to the extra-cellular site [9, 10]. Preliminary reports have suggested improved performance for PSMA PET over conventional imaging in detecting RCC metastases [11, 12].

Therefore, this study aimed to assess the clinical impact of PSMA PET on the management of patients with recurrent or de-novo metastatic RCC when used across multiple institutions.

## Methods

### Study design and patient population

A multicentre retrospective study was performed across three tertiary hospitals (Royal Brisbane Women’s Hospital, Princess Alexandra Hospital and Wesley Hospital). Inclusion criteria were adult patients who underwent PSMA PET during the period of January 2015 to June 2020 for restaging of resected RCC with suspected recurrence on conventional imaging or initial staging of metastatic RCC. Patients were excluded if PSMA PET was performed for localised RCC or other cancers. This study received ethics and governance approval (Reference numbers HREC/2020/QRBWH/64912; UCH 2020.19.329)

### Imaging

Siemens Biograph PET scanner (Siemens AG, Munich, Germany) were used at two sites (Royal Brisbane and Women’s Hospital and Princess Alexandra Hospital), while either Philips Ingenuity TF 128 slice PET/CT scanner (Phillips Healthcare, Amsterdam, Netherlands) or GE Discovery MI DR PET CT scanner (GE Healthcare, Chicago, USA) was used at one site (Wesley Hospital).

All sites used [^68^Ga]Ga-PSMA-11 initially, with two sites (Royal Brisbane and Women’s Hospital and Princess Alexandra Hospital) migrating to use of [^18^F]F-PSMA-1007 due to lower renal excretion and preference. Imaging were performed from skull vertex to the upper thighs. [^68^Ga]Ga-PSMA-11 PET images were obtained following intravenous delivery of average 145 MBq (Range: 100-162 MBq) and an average uptake time of 62 minutes (Range: 35-98 minutes). [^18^F]F-PSMA-1007 PET images were obtained following intravenous delivery of average 242 MBq (Range: 163-296 MBq) and an average uptake time of 120 min (Range: 57-169 minutes). Low dose CT was also performed for anatomical correlation at Royal Brisbane and Women’s Hospital and Princess Alexandra Hospital, while diagnostic CT was performed at Wesley Hospital. PET/CT Maximum intensity projection (MIP) images are shown in Supplementary Fig. 1, illustrating the differences in normal physiological distribution between the two tracers. Lesions were considered suspicious for metastasis when PSMA uptake was greater than background physiological uptake in a typical distribution for metastatic disease, with anatomical correlation on CT. More complex cases (e.g., solitary metastatic disease, or an unusual metastatic site) were discussed at multidisciplinary meetings for further evaluation, including additional imaging or biopsy. Images were interpreted by an experienced nuclear medicine specialist, while diagnostic CT was similarly interpreted by an experienced radiologist.

### Data collection

PSMA PET databases at each institution were generated and queried for RCC patients with recurrent or de-novo metastatic disease. All available electronic medical records were accessed for data collection. Demographic data included age, sex, primary tumour histology (including variant morphology, such as sarcomatoid or rhabdoid differentiation) and line of systemic therapy prior to PSMA PET. Haemoglobin, neutrophil, platelet count, corrected calcium, Karnofsky performance status (≥80% or <80%) and time to systemic therapy were recorded for patients with metastatic disease. International metastatic renal cell carcinoma database consortium (IMDC) prognostic score at time of systemic therapy, or at time of PSMA PET (if not receiving systemic therapy) was calculated [13].

PSMA PET reports were reviewed and sites of disease and maximum standardized uptake (SUVMax) values were recorded. Diagnostic CT reports were reviewed, and sites of disease recorded if performed within 4 weeks prior to PSMA PET. Concordant findings were defined as the same sites of disease identified on PSMA PET and CT. Dis-concordant findings were defined as new sites of disease identified on PSMA PET, not identified on diagnostic CT, or PSMA PET not showing avidity in suspected metastatic sites on diagnostic CT. Histopathology of biopsied PSMA avid sites were also recorded if performed.

### Disease classification

Patients were grouped into either 1) recurrent disease, where PSMA PET scan was performed to delineate diagnosis and management of suspected local or distant recurrence on surveillance conventional imaging post nephrectomy for RCC. 2) In the initial or subsequent management of de-novo metastatic disease.

Oligometastatic disease was defined as less than 5 metastatic sites that were amenable to definitive surgery or radiation.

### Outcomes

The primary outcome of this study was to determine the change in clinical management following PSMA PET. Change in management was classified as intensification (change in modality of treatment, change in line of treatment or treatment to different site), or de-intensification (surveillance or biopsy rather than systemic therapy or resection). Each medical record was reviewed by two independent investigators for data confirmation, including consensus on management before and after PSMA PET, with a difference of opinion moderated by a third independent investigator.

The secondary outcomes were histological correlation of PSMA avid sites with PET parameters [SUVmax], comparison of PSMA PET to diagnostic CT, and time to systemic treatment in patients who underwent surveillance or local treatment [surgery or radiation] based on PSMA PET.

### Statistical analysis

Data was managed with Microsoft Excel. Management plans before and after PSMA PET are illustrated via Sankey diagram which was generated via Microsoft Excel. Baseline characteristics were compared using Fisher exact test. *P* value <0.05 was considered significant. Kaplan Meier time to event analysis was used to calculate median time to treatment. STATA v17 software was used for the above analysis.

## Results

**Fig. 1:**
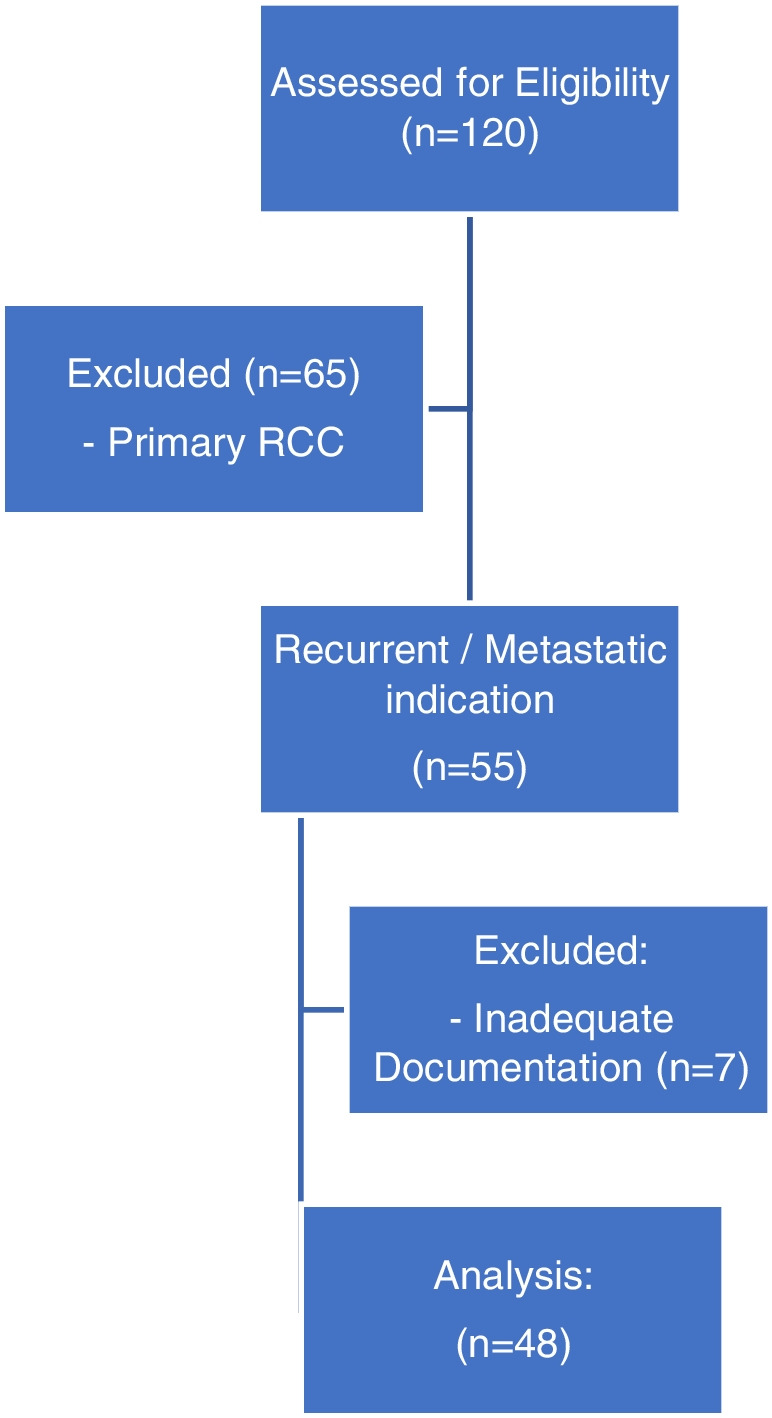
Study profile

120 patients were screened for eligibility, of which 51 PSMA PET scans in 48 patients were included for analysis. Patients were excluded because PSMA PET was performed for primary staging or similar or inadequate documentation (Fig. 1). 35 patients (35/48, 73%) underwent [^68^Ga]Ga-PSMA-11, while 13 patients (13/48, 27%) underwent [^18^F]F-PSMA-1007. 35 patients (35/48, 73%) had suspected recurrent locally or distant metastatic disease, while 13 patients (13/48, 27%) had de-novo metastatic disease. Median age was similar between recurrent and de-novo metastatic groups [64 v 63 years]. Primary tumour was predominantly clear cell RCC in both groups [100% v 92% *p*=0.27]. PSMA PET was positive in 86% (44/51) of scans. A higher proportion of favourable IMDC risk was observed in the recurrent group (60% v 23%; *p*=0.05), and lower proportion of intermediate IMDC risk [26% v 62% *p*=0.04), likely driven by short interval (less than 1 year) from diagnosis to treatment (11% vs 62%; *p*<0.01), and thrombocytosis (0% v 23% *p*=0.02). The de-novo metastatic group had a higher proportion of previous systemic therapy use (31% vs 3%, *p*=0.02), which was predominantly Tyrosine kinase inhibitor (77% vs 31%, *p*=<0.01). Demographics are outlined in Table 1.

**Table 1:** Demographics

	Recurrent	De-novo Metastatic	p
	n (%)	n (%)	
n	35	13	
Sex			
M	24 (69)	8 (62)	0.74
F	11 (31)	5 (38)	0.74
Median Age (yrs)	64 (Range: 45-74)	63 (Range: 47-85)	
Previous Nephrectomy	35 (100)	5 (38)	**<0.01**
Primary Tumour			
Clear cell	35 (100)	12 (92)	0.27
Unclassified	0 (0)	1 (8)	0.27
Sarcomatoid differentiation	2 (6)	1 (8)	1
Rhabdoid differentiation	1 (3)	1 (8)	1
IMDC			
Low Hb	3 (9)	0 (0)	0.55
Neutrophilia	1 (3)	3 (23)	0.06
Hypercalcaemia	0 (0)	1 (8)	0.27
Thrombocytosis	0 (0)	3 (23)	**0.02**
KPS <80%	3 (9)	2 (15)	0.6
Diagnosis to Treatment <1yr	4 (11)	8 (62)	**<0.01**
Favourable	21 (60)	3 (23)	**0.05**
Intermediate	9 (26)	8 (62)	**0.04**
Poor	0 (0)	2 (15)	0.07
Not available/applicable	5 (14)	0 (0)	0.3
Prior systemic therapy to PSMA PET			
Yes	1 (3)	4 (31)	**0.02**
1L of systemic therapy			
TKI	11 (31)	10 (77)	**<0.01**
Immunotherapy	8 (23)	1 (8)	0.41
No systemic therapy to date	16 (46)	2 (15)	0.09

Overall, there was a change in management in 14 patients (14/48, 29%), with intensification observed for 11 patients (11/48, 23%) and de-intensification observed for 3 patients (3/48, 6%) due to PSMA PET.

### Management change - Recurrent disease

**Fig. 2:**
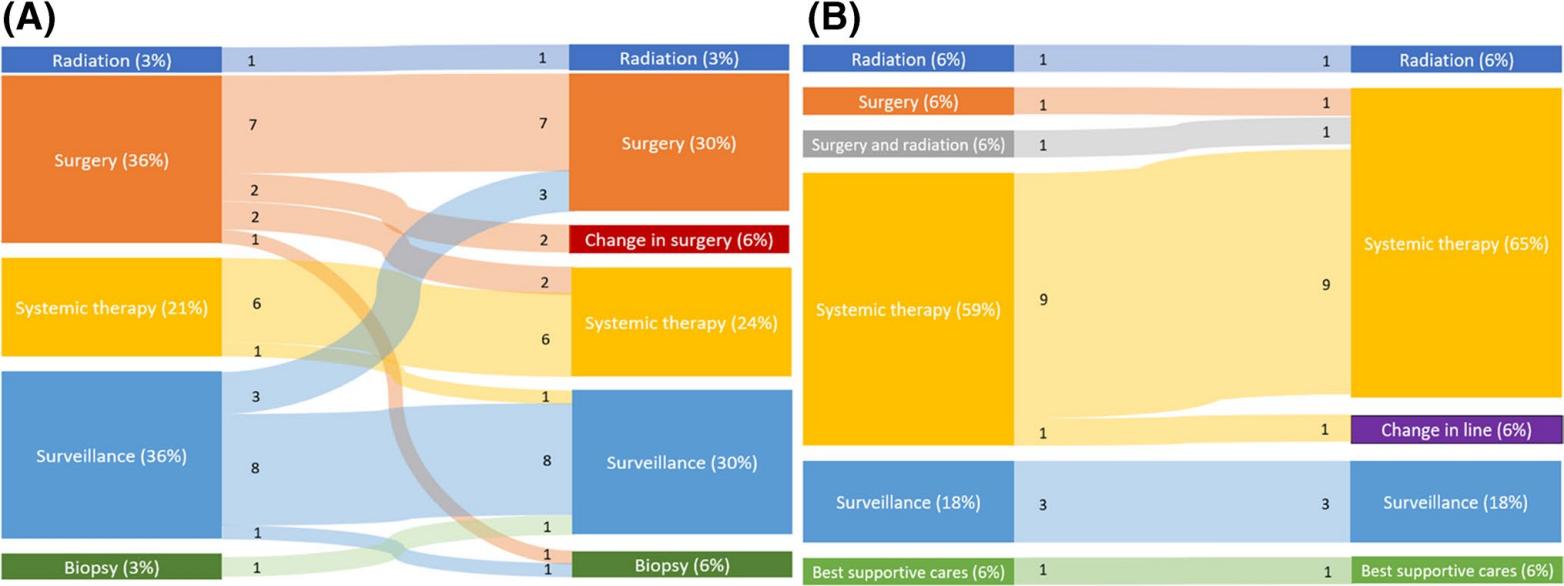
Sankey diagrams representing treatment before and after PSMA PET in the recurrent disease group (**A**) and metastatic disease group (**B**)

37 PSMA PET scans were performed in 35 patients for suspected recurrent disease on conventional imaging. Change in management was observed for 11 patients (11/35, 31%), grouped according to pre- and post-PET pathways (Fig. 2a) with details included in Table 2.

**Table 2:** details of change of management as a result of PSMA PET for patients in recurrent and metastatic groups

Patient No	Pre-PSMA Plan	Post PSMA Plan	Comment
Recurrent disease			
1 + 2	Surveillance	Adrenalectomy	Adrenal metastasis better delineated on PSMA PET v CT
3	Surveillance	Iliac node dissection	Iliac node metastasis better delineated on PSMA PET v CT
4	Surveillance	Biopsy of pulmonary nodule.	Pulmonary metastasis better delineated on PSMA PET v CT
5	Biopsy of mediastinal node	Surveillance	No PSMA avidity of enlarged mediastinal node on CT.
6	TKI*	Surveillance	Borderline PSMA avidity of enlarged mediastinal node on CT
7	RPLND**	Biopsy of RPLN***	No PSMA avidity of enlarged RPLN on CT.
8	Resection abdominal wall recurrence	TKI	More extensive recurrence identified on PSMA PET v CT, determined to be unresectable.
9	Adrenalectomy	Immunotherapy	Poly-metastatic disease identified on PSMA PET – pulmonary and bone metastasis
10	Resection of local recurrence	Resection of local recurrence and hemi-hepatectomy	Liver metastasis identified on PSMA PET occult on CT.
11	Resection of local recurrence	Change in surgical approach	Change in surgical approach required for more extensive local recurrence.
De-novo Metastatic disease			
1	RPLND**	Systemic Therapy	More extensive RPLN*** detected on PSMA PET v CT, deemed unresectable.
2	Resection of local recurrence and radiation to L1 metastasis.	Systemic Therapy	Poly-metastatic disease identified on PSMA PET - Pulmonary, bone and liver metastasis noted.
3	TKI*	Check-point inhibitor	Gastric metastasis identified on PSMA PET not identified on CT consistent with progression disease.

*Tyrosine kinase inhibitor **retroperitoneal lymph node dissection ***retroperitoneal lymph node

Treatment or investigation intensification after PSMA PET was observed for 8 patients (8/35, 23%). We observed conversion from initial surveillance to resection or biopsy (4/35, 11%) because of PSMA avidity at suspected sites that were equivocal on CT scan, or more extensive surgery for recurrence (2/35, 6%), when PSMA PET identified a greater burden of disease. Conversion from resection or radiation for oligometastatic disease based on CT to systemic therapy (2/35, 6%) occurred due to poly-metastatic disease being identified on PSMA PET.

De-intensification of treatment or investigation after PSMA PET was observed for 3 patients (3/35, 9%) due to a lack of avidity in a suspected site of recurrence on CT, from initial systemic therapy (1/35, 3%), resection (1/35, 3%) and biopsy (1/35, 3%).

Although management was not altered, PSMA PET influenced management by confirming oligometastatic disease in 7 patients (7/35, 20%), proceeding with surgery or radiation as planned. In 2 patients (2/35, 6%) a lack of PSMA avidity of suspected recurrence site confirmed management of surveillance.

### Management change – De-novo metastatic disease

A total of 14 PSMA PET scans were performed in the de-novo metastatic setting in 13 patients. Change in management was observed for 3 patients (3/13, 23%), grouped according to pre- and post-PET pathways (Fig. 2b) with details included in Table 2.

All 3 patients had intensification of treatment, with two patients proceeding to systemic therapy instead of oligometastatic therapy, when unresectable or poly-metastatic disease was identified on PSMA PET. One patient had a change in line of systemic therapy when a new gastric metastasis was identified on PSMA PET which was not evident on CT.

PSMA PET provided confirmation of oligometastatic progression identified on CT (1/13, 8%), which proceeded with planned radiation to a right hilar node, while the same line of systemic therapy was continued in 2 patients based on PSMA PET findings.

#### Imaging comparison

**Fig. 3:**
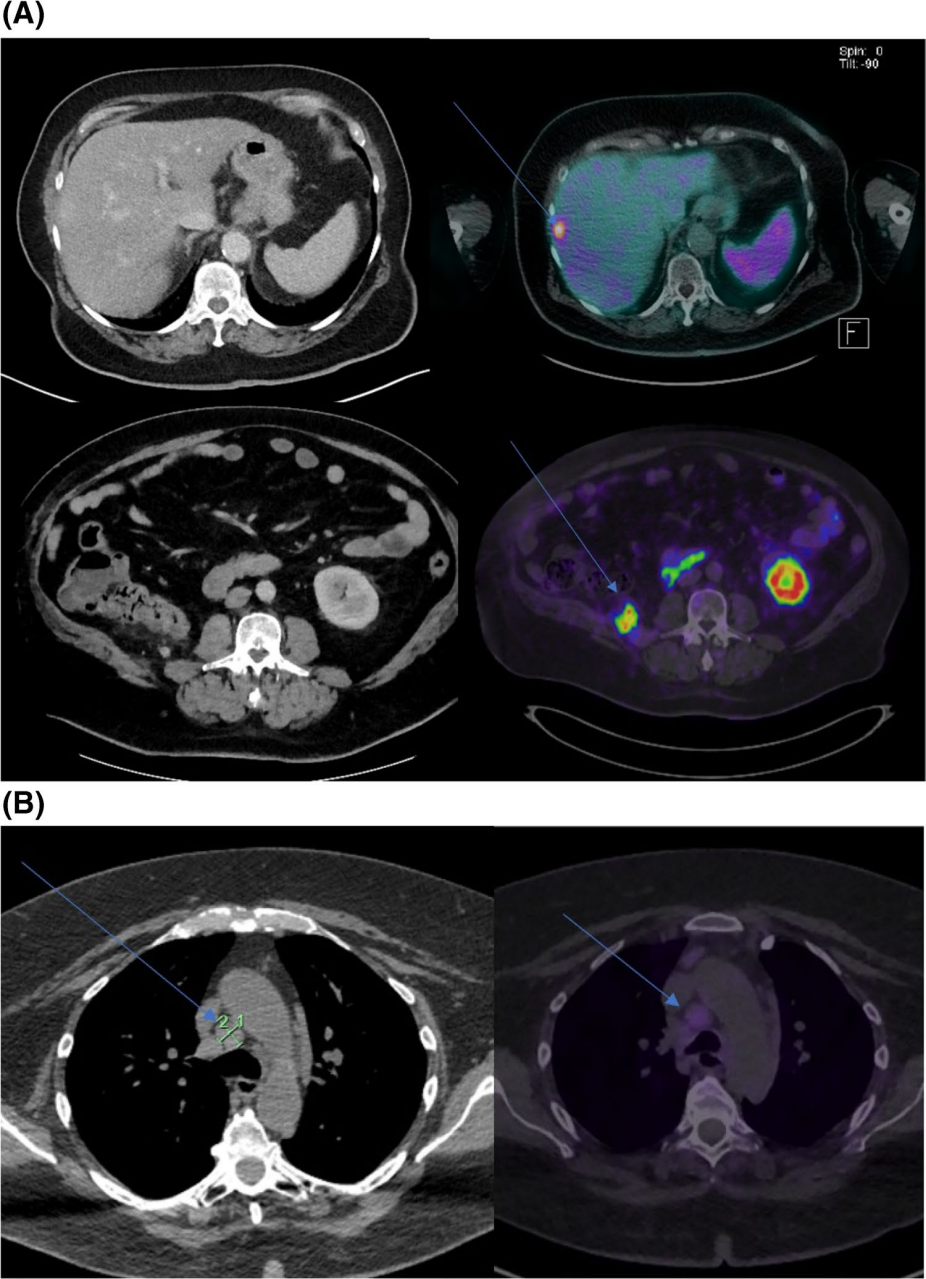
Comparison of PSMA PET compared to CT. **A** shows better delineation of metastatic disease than CT, **B** refuted suspected metastatic disease on CT

Corresponding diagnostic CT within one month prior to PSMA PET was available for 27 patients. The majority of CT scans were discordant with PSMA PET (17/27, 63%), mostly due to PSMA PET highlighting more sites of disease (*n*=14; Fig. 3a), including sub-diaphragmatic nodal disease (18.5%), bone metastasis (14.8%) and adrenal metastasis (14.8%; Supplementary Table 1). PSMA PET was discordant with CT scan findings (*n*=3; Fig. 3b), due to lack of avidity, in pulmonary metastasis (7.4%) and mediastinal nodal disease (3.7%).

[^68^Ga]Ga-PSMA-11 was performed for 17 patients (17/27, 63%), with PSMA PET highlighting more sites of disease in comparison to CT in 9 patients (9/17, 53%). [^18^F]F-PSMA-1007 was performed for 10 patients (10/27, 37%) and identified more sites of disease in 50% (5/10) of patients. All 3 patients who had discordant findings due to lack of avidity on PSMA PET received [^18^F]F-PSMA-1007.

For patients with recurrent disease, 25 patients (25/35, 71%) underwent [^68^Ga]Ga-PSMA-11, while 10 patients (10/35, 29%) underwent [^18^F]F-PSMA-1007 in this cohort. Percentage of patients requiring change in management was similar in both groups [28% vs 30% *p*=1.0]. For patients with de-novo metastatic disease, 10 patients (10/13, 77%) underwent [^68^Ga]Ga-PSMA-11, while 3 patients (3/13, 23%) underwent [^18^F]F-PSMA-1007 in this subgroup. All 3 patients who had a change in management received [^68^Ga]Ga-PSMA-11 [30% vs 0% *p*=0.53].

### Histopathological correlation with imaging

Biopsy of PSMA avid sites occurred for 30 patients, of which most (28/30, 93%) were clear cell renal carcinoma (2 of these had sarcomatoid differentiation). Other morphologies included carcinoid (1/30, 3%, [^18^F]F-PSMA-1007) and urothelial carcinoma (1/30, 3%, [^68^Ga]Ga-PSMA-11). Two patients (2/30; 6%, [^18^F]F-PSMA-1007 and [^68^Ga]Ga-PSMA-11) proceeded to biopsy/resection of non-PSMA avid site due to suspected recurrence on CT, both returned benign histology. Median SUVmax of patients with clear cell renal carcinoma was 14 (IQR: 8.35-23.3). The median SUVmax did not differ for clear cell renal carcinoma between [^68^Ga]Ga-PSMA-11 and [^18^F]F-PSMA-1007 tracer [14 vs 14.4]. The median SUVmax and histology is located in Table 3.

**Table 3:** Histopathology of biopsied PSMA avid sites

Histopathology	n	%	Median (SUVMax)	IQR
Clear cell*	26	86.7%	14	8.35-23.2
Sarcomatoid*	2	6.7%	25.2	25.2-25.2
Urothelial	1	3.3%	8.3	8.3-8.3
Carcinoid	1	3.3%	2.7	2.7-2.7

*SUVMax for 1 clear cell [sternum] and 1 sarcomatoid renal cell [sternum] were not available and excluded from calculations

### Oncological outcomes – Recurrent disease

Patients with oligometastatic recurrent disease based on PSMA PET (14/35, 40%) were treated with local therapies, such as surgical resection (12/35, 34%) and radiation (2/35, 6%). During the follow-up (median 36 months, IQR 16-68), 4 patients (4/35, 11%) required systemic therapy after local therapy with a median time to commencement not reached [IQR: 52- NR months]. 2 patients (2/35, 6%) started systemic therapy within 12 months. 2 patients died from non-RCC causes before requiring systemic therapy.

During the follow-up (median 47 months, IQR: 34-55) of patients with suspected recurrence on CT who underwent surveillance based on PSMA PET results (10/35,29%), 5 patients (5/35, 14%) commenced systemic therapy. Median time to starting systemic therapy from PSMA PET was 36.1 months (IQR 21-49), with 1 patient requiring systemic therapy within 12 months. 1 patient died from non-RCC cause. 1 patient died from RCC cause who declined systemic therapy. Median time to starting systemic therapy was numerically longer for patients with negative PSMA PET in comparison to positive [36 v 21.1months *p*=0.28] but did not reach statistical significance.

## Discussion

PSMA PET has the potential to improve precision in RCC staging and appropriateness of local or systemic therapy. Here, we observed that PSMA PET performed in patients with recurrent or de-novo metastatic RCC changed management in 29% (14/48), which was numerically higher for patients with recurrent than de-novo metastatic disease (31% vs 23%, *p*=0.73). Management plans were more likely to be intensified than de-intensified (23% vs 6%) due to additional or absent PSMA activity, respectively. Despite initial de-intensification, some patients still progressed to require systemic therapy (*n*=5) with variable interval treatment period. This is consistent with the natural history of RCC, with variable and late recurrences noted. The percentage of patients who required change in management was similar despite the tracer used.

Other centres have reported higher impacts, such as Udovicich and colleagues who reported management change for 49% [14]. Their cohort had a higher proportion of oligometastatic disease considered for local therapies based on conventional imaging compared to the current cohort (72% vs 33%, respectively), where management is more likely to change according to the current dataset (44%). The management impact is likely to be most significant for the oligometastatic cohort, with potential for change to either systemic therapy or surveillance based on either more or less extensive disease noted on PSMA PET, respectively. Correct characterisation of disease is important to avoid potentially unnecessary morbidity (e.g. surgery), which was outlined in a series of 14 patients with oligometastatic clear cell RCC based on CT, where 21.4% had poly-metastatic disease when evaluated with PSMA PET [15]. Conversely, accurate and earlier identification of oligometastatic disease with PSMA PET may prompt earlier local therapy (3/51, 6% in this study) and delay the initiation of systemic therapy post local therapy, shown here with 57% (8/14) of patients alive and not requiring systemic therapy to date, with a median time to systemic treatment not reached.

CT with intravenous contrast is the current standard of care for the staging of RCC. Limitations of CT include inability to distinguish between reactive or metastasis in enlarged lymph nodes and, difficulty in detecting small volume local recurrence and metastases. Several case series have highlighted the improved ability of PSMA PET to detect metastasis, including nodal disease too small to be characterised on CT and bone metastasis [16]. A summary of these studies is outlined in Supplementary Table 2. Conversely, a negative PSMA PET may be useful to rule out recurrence despite CT findings, indicated here by both management change patterns and negative histopathology of non-avid PSMA sites. While some patients with negative PSMA PET progressed to systemic therapy, 43% of patients in this subgroup are alive and have not required systemic therapy to date. Other molecular imaging techniques, such as FDG PET, may complement PSMA PET for improving precision of staging [17], while PSMA PET may assist with local staging (including tumour thrombus characterisation) [18].

In addition to precise staging, molecular imaging techniques may assist with assessing treatment response to systemic therapy in metastatic RCC. We found that, despite stable disease on CT, PSMA PET indicated progressive disease prompting change in systemic therapy for 1 patient, while partial responses were found in 2 patients, with one ceasing immunotherapy due to toxicity. These findings are supported by an observational study by Mittlemier and colleagues, where 9 out of 11 patients had stable disease after systemic therapy per CT assessment, however PSMA PET showed that most (6 out of 9 patients) had either complete or partial response and 1 patient had progressive disease [19].

The strengths of this study include the multi-centre, real-world data including multiple tracers and is one of the largest available series of PSMA PET. The larger number of biopsies or resections performed on PSMA avid sites for histopathological correlation was also a strength. Histopathological correlation of all PSMA avid sites is not appropriate in clinical practice, but was mitigated by assessment of atypical cases, such as solitary sites or equivocal diagnostic CT findings. The limitations include the retrospective nature and biased patient selection for PSMA PET imaging due to suspicion on CT, as well as different tracers used which may affect PSMA ligand avidity for non-biological reasons [20]. Retrospective determination of change in management is a limitation, but was mitigated through use of multiple, independent assessors.

In conclusion, PSMA PET provided clinically useful information in addition to conventional imaging, with high pathological concordance, to result in change in management for 29% of patients. These preliminary data support potential benefit of PSMA PET in renal cancer staging, both for accurate disease characterisation and potential response to treatment. Prospective trials are encouraged to further investigate the prognostic and predictive characteristics of PSMA PET for advanced RCC.

### Supplementary information

ESM 1 (DOCX 156 kb)

## Data Availability

The datasets generated during and/or analysed during the current study are not publicly available in order to protect patient privacy. The corresponding author may provide anonymised data on reasonable request.
