# Supplementary material for: Clinical impact of Prostate-Specific Membrane Antigen Positron Emission Tomography (PET) on intensification or deintensification of advanced renal cell carcinoma management
Source: Eur J Nucl Med Mol Imaging. 2023 Aug 18;51(1):295–303. doi: 10.1007/s00259-023-06380-4 (PMC10684606; doi:10.1007/s00259-023-06380-4)
Supplement: Supplementary file 1 — (DOCX 156 kb) [file 259_2023_6380_MOESM1_ESM.docx]

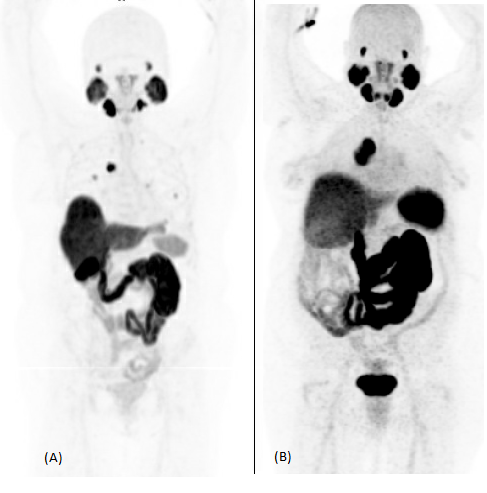


Suppementary Figure 1: A) Shows PSMA PET utilising [^18^F]F-PSMA-1007 for patient with recurrent RCC with pulmonary metastasis. The scan shows normal distribution of the tracer with no urinary bladder uptake due to non-urinary secretion. B) Shows PSMA PET utilising [^68^Ga]Ga-PSMA-11 tracer of patient with recurrent RCC with right hilar metastasis. Scan shows normal distribution including urinary bladder uptake due to urinary excretion of tracer.

|  | Better Delineated | | Refuted | |
| --- | --- | --- | --- | --- |
| Sites | n | % | n | % |
| Infra-diaphragmatic nodal | 5 | 18.5% | 0 | 0.0% |
| Adrenal | 4 | 14.8% | 0 | 0.0% |
| Bone | 4 | 14.8% | 0 | 0.0% |
| Supra-diaphragmatic nodal | 3 | 11.1% | 1 | 3.7% |
| Abdominal wall | 2 | 7.4% | 0 | 0.0% |
| Local recurrence | 2 | 7.4% | 0 | 0.0% |
| Pulmonary | 1 | 3.7% | 2 | 7.4% |
| Gastric | 1 | 3.7% | 0 | 0.0% |
| Thyroid | 1 | 3.7% | 0 | 0.0% |
| Small Bowel | 1 | 3.7% | 0 | 0.0% |

Supplementary Table 1: Sites of disease better delineated on PSMA PET

| Author (Year) | n | Tracer | Comparator | Histology | Comments |
| --- | --- | --- | --- | --- | --- |
| Rowe (2015) [14] | 5 | 18F-DCFPyL | CT +/- MR | ccRCC | PSMA identified more sites of disease. Unable to detect a small liver lesion, likely due to size and background activity |
| Rhee (2016) [10] | 10 | 68Ga-PSMA-11 | CT +/- MR/USS | ccRCC (n=8)  pRCC (n=1)  uRCC (n=1) | PSMA identified more sites of mRCC. Sensitivity improved from 68.7% to 92.11%. Positive predictive value improved from 80% to 97.22%. |
| Sawicki (2017) [17] | 6 | 68Ga-PSMA-11 | Nill | ccRCC (n=4)  pRCC (n=1)  chRCC (n=1) | Useful in detecting metastatic ccRCC. No additional diagnostic value in detecting primary RCC due to background PSMA uptake in kidney. |
| Siva (2017) [18] | 8 | 68Ga-PSMA-11 | FDG | ccRCC (n=7) pRCC (n=1) | ccRCC metastasis typically more PSMA avid than FDG avid. Locally recurrent pRCC not PSMA avid. |
| Myer (2019) [13] | 14 | 18F-DCFPyL | CT +/- MR | ccRCC | PSMA PET identified more lesion in 28.6% of patients, and 21.4% were no longer considered oligometastatic. |
| Yin (2019) [19] | 8 | 18F-DCFPyL | CT +/- MR | pRCC (n=3) chRCC (n=2) uRCC (n=2)  Xp translocated RCC (n=1) | 13.7% of non-clear cell RCC showed PSMA-uptake suggesting it is not an appropriate modality in this sub-group. |
| Liu (2020) [20] | 15 | 18F-DCFPyL | FDG | ccRCC | PSMA PET improved detected of local recurrence and bone metastasis. |
| Mittlemeir (2021) [16] | 11 | 18F-DCFPyL | CT | ccRCC | PSMA PET may better delineate response to TKI in comparison to CT. |
| Tariq  (2022) [15] | 11 | 18F-DCFPyL  68Ga-PSMA-11 | FDG | ccRCC (n=10)  uRCC (n=1) | PSMA PET outperformed FDG PET in 18.2% of patients |
| Udovivich  (2022) [12] | 61 | 18F-DCFPyL  68Ga-PSMA-11 | CT +/- FDG | ccRCC (n=54)  chRCC (n=2)  pRCC (n=2)  cRCC+pRCC (n=2)  Unclassified (n=2) | Change in management in 49% of patients due to PSMA PET Identified more lesions than CT in 25%, and less lesions in 26% |

Supplementary Table 2: Summary of literature review assessing PSMA PET in RCC in comparison to SOC imaging. *ccRCC = clear cell renal carcinoma **pRCC = papillary renal cell carcinoma ***uRCC = unclassified renal cell carcinoma ****chRCC = chromophobe renal cell carcinoma
